# Doxorubicin conjugated with a trastuzumab epitope and an MMP-2 sensitive peptide linker for the treatment of HER2-positive breast cancer

**DOI:** 10.1080/10717544.2018.1435746

**Published:** 2018-02-06

**Authors:** Yiwen You, Zhiyuan Xu, Yun Chen

**Affiliations:** a School of Pharmacy, Nanjing Medical University, Nanjing, China;; b State Key Laboratory of Reproductive Medicine, Nanjing, China

**Keywords:** Doxorubicin-peptide conjugate, trastuzumab epitope, MMP-2 sensitive peptide linker, HER2-positive breast cancer, multiple targets

## Abstract

HER2-positive breast cancer correlates with more aggressive tumor growth, a poorer prognosis and reduced overall survival. Currently, trastuzumab (Herceptin), which is an anti-HER2 antibody, is one of the key drugs. There is evidence indicating that conjugation of trastuzumab with chemotherapy drugs, such as doxorubicin (DOX), for multiple targets could be more effective. However, incomplete penetration into tumors has been noted for those conjugates. Compared to an antibody, peptides may represent an attractive alternative. For HER2, a similar potency has been observed for a 12-amino-acid anti-HER2 peptide mimetic YCDGFYACYMDV-NH_2_ (AHNP, disulfide-bridged) and full-length trastuzumab. Thus, a peptide, GPLGLAGDDYCDGFYACYMDV-NH_2_, which consists of AHNP and an MMP-2 cleavable linker GPLGLAGDD, was first designed, followed by conjugation with DOX *via* a glycine residue at the N-terminus to form a novel DOX-peptide conjugate MAHNP-DOX. Using HER2-positive human breast cancer cells BT474 and SKBR3 as *in vitro* model systems and nude mice with BT474 xenografts as an *in vivo* model, this conjugate was comprehensively characterized, and its efficacy was evaluated and compared with that of free DOX. As a result, MAHNP-DOX demonstrated a much lower *in vitro* IC_50_, and its *in vivo* extent of inhibition in mice was more evident. During this process, enzymatic cleavage of MAHNP-DOX is critical for its activation and cellular uptake. In addition, a synergistic response was observed after the combination of DOX and AHNP. This effect was probably due to the involvement of AHNP in the PI3K–AKT signaling pathway, which can be largely activated by DOX and leads to anti-apoptotic signals.

## 1. Introduction

Overexpression of the human epidermal growth factor receptor family member HER2, which is also known as erbB-2/neu, is found in approximately 20–30% of breast cancer cases (i.e. HER2-positive breast cancer) and correlates with more aggressive tumor growth, poorer prognosis and reduced overall survival (Tai et al., 2010; Dent et al., 2013; Rimawi et al., 2015). Similar to its other family members, HER2 has the following three domains: extracellular ligand-binding, transmembrane and intracellular tyrosine kinase domains (Tai et al., 2010; Rimawi et al., 2015). These domains are essential for receptor dimerization and downstream signaling, which subsequently lead to cell proliferation and cell survival (e.g. the PI3K/AKT pathway) and ultimately contribute to breast cancer risk (Harari & Yarden, 2000; Rimawi et al., 2015).

Currently, trastuzumab (Herceptin) is one of the key drugs in the treatment of HER2-positive breast cancer (Tokuda et al., 2009; Baselga, 2010). This humanized monoclonal antibody can bind to the extracellular segment of HER2 and prevent its dimerization, which leads to inhibition of HER2-mediated malignant transformation (Le et al., 2003; Ménard et al., 2003; Gemmete & Mukherji, 2011). Some of the therapeutic effects may also be due to the downregulation of HER2 and the increase in turnover, which results in a decrease in signaling from HER2 (Wonders & Reigle, 2009). In addition, there is evidence indicating that combination of trastuzumab with chemotherapy drugs, such as doxorubicin (DOX), could be more effective (Tokuda et al., 2009; Rimawi et al., 2015). In principle, DOX enters cells *via* simple diffusion and intercalates into DNA, which inhibits topoisomerase II and prevents DNA replication (Hortobágyi, 1997; Wonders & Reigle, 2009). Despite its observed clinical efficacy, such a simple drug combination is generally not recommended due to low selectivity and severe cardiotoxicity coming from the cumulative effects of both trastuzumab and DOX (Wonders & Reigle, 2009, Rochette et al., 2015). In this context, a trastuzumab-DOX conjugate has been recently developed and provides improved selectivity in drug delivery and reduced cardiotoxicity by relying on antibody trastuzumab-mediated specific recognition of the tumor antigen HER2 (Tarcic & Yarden, 2013; Zhang et al., 2013). However, the trastuzumab-DOX conjugate has had incomplete penetration of tumors due to the “binding site barrier” effect (Aina et al., 2002; Graff & Wittrup, 2003; Thurber et al., 2008), which is consistent with the results for most other antibody-drug conjugates (ADC).

Compared to an antibody, a peptide may represent an attractive alternative because of its small molecular weight, excellent tissue/cell penetration, easy production, chemical conjugation flexibility and low probability of undesirable immunogenicity (Aina et al., 2002; Tai et al., 2010). For HER2, a 12-amino-acid anti-HER2 peptide mimetic YCDGFYACYMDV-NH_2_ (AHNP, disulfide-bridged), derived from the complementarity-determining region (CDR) heavy chain 3 (H3) of trastuzumab can specifically bind to the extracellular domain of HER2 with high affinity (Park et al., 2000; Berezov et al., 2001; Tan et al., 2006). More importantly, similar potency has been observed for AHNP and full-length trastuzumab (Park et al., 2000).

**Figure 1.**
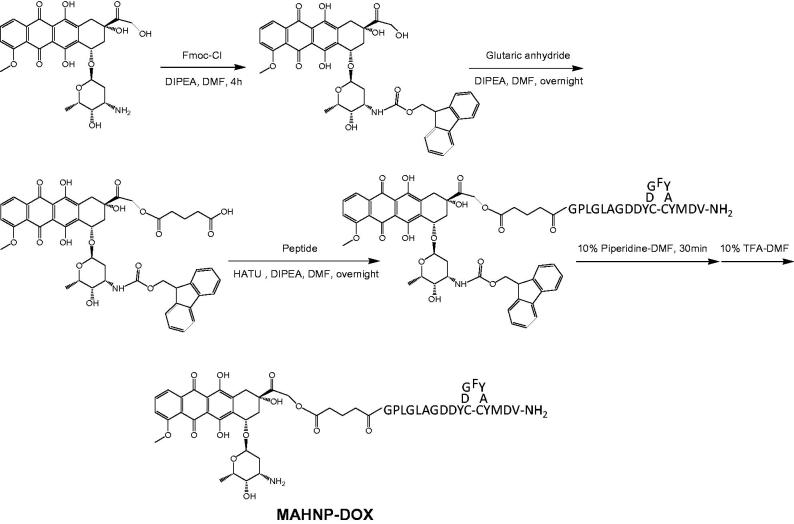
Structure of MAHNP-DOX and its synthetic scheme.

In this study, a peptide GPLGLAGDDYCDGFYACYMDV-NH_2_, which consists of a cleavable linker GPLGLAGDD and the anti-HER2 peptide AHNP, was designed and then conjugated with DOX *via* a glycine residue at the N-terminus to form a novel DOX-peptide conjugate MAHNP-DOX (Figure 1). The linker component PLGLAG is predominantly sensitive to an enzyme called matrix metalloproteinase 2 (MMP-2), and this enzyme-sensitive substrate strategy was employed for tumor targeting of DOX (Shi et al., 2012; Torchilin 2014; Zhu et al., 2014). Using the HER2 positive human breast cancer cells BT474 and SKBR3 as *in vitro* model systems and nude mice with BT474 xenografts as an *in vivo* model, this conjugate was comprehensively characterized, and its efficacy was evaluated and compared to free DOX. In addition, the involvement of the PI3K–AKT signaling pathway and cell cycle arrest in MAHNP-DOX activity were also explored.

## 2. Experimental

### 2.1. Chemicals and reagents

Peptide GPLGLAGDDYCDGFYACYMDV-NH_2_ was developed by ChinaPeptides Co., Ltd. (Shanghai, China). Doxorubicin hydrochloride (DOX·HCl) was purchased from Hisun Pharmaceutical Co., Ltd. (Zhejiang, China). MMP-2 Inhibitor I, a selective inhibitor of MMP-2, was purchased from Santa Cruz Biotechnology, Inc. (Santa Cruz, CA, USA). Glutaric anhydride, 1-[bis(dimethylamino)methylene]-1*H*-1,2,3-triazolo[4,5-b]pyridinium 3-oxid hexafluorophosphate (HATU), diethyl ether, ethyl acetate and trifluoroacetic acid (TFA) were obtained from Aladdin Industrial Corporation (Shanghai, China). 3-(4,5-Dimethyl-2-thiazolyl)-2,5-diphenyl-2-*H*-tetrazolium bromide (MTT), d-(+)-mannitol, *N*-(2-hydroxyethyl) piperazine-*N′*-(2-ethanesulfonic acid) (HEPES), piperidine and McCoy’s 5 A Modified Medium were purchased from Sigma-Aldrich (St. Louis, MO, USA). Trypan blue was obtained from Generay Biotech Co., Ltd (Shanghai, China). RPMI 1640 Medium and Dulbecco’s Modified Eagle Medium (DMEM) and fetal bovine serum (FBS) were obtained from Invitrogen (Burlington, ON, Canada). Mammary epithelial cell growth medium (MEGM) was obtained from LONZA (Basel, Switzerland). Penicillin/streptomycin solution was supplied by HyClone Laboratories, Inc. (Logan, UT, USA). Phosphate buffered saline (PBS) was from Beyotime Institute of Biotechnology (Jiangsu, China). *N,N*-dimethylformamide (DMF), *N,N*-diisopropylethylamine (DIPEA), ethylenediaminetetraacetic acid (EDTA), potassium hydroxide, sodium bicarbonate, 9-fluorenylmethyl chloroformate (Fmoc-Cl) and hydrochloric acid were obtained from Sinopharm Chemical Reagent Company (Shanghai, China). Acetonitrile (ACN) and methanol were HPLC grade and were purchased from ROE Inc. (Newark, NJ, USA). Formic acid (FA) was provided by Xilong Chemical Industrial Factory Co., Ltd (Shantou, China). Water was purified and deionized using a Milli-Q system from Millipore (Bedford, MA, USA).

### 2.2. Preparation of peptide-conjugated DOX

The synthesis of MAHNP-DOX was carried out according to the previously reported procedure (Nagy et al., 1996). The detail is shown below.

#### 2.2.1. Synthesis of *N*-fmoc-DOX

Fmoc protection of amines has been previously described in a number of studies (Myers et al., 1997; Hill et al., 2006; Perron et al., 2009). In this study, 29 mg (0.05 mmol) DOX was dissolved in 4 mL of DMF and stirred at room temperature under nitrogen atmosphere for 10 min. Then, 1 mL of Fmoc-Cl (26 mg, 0.1 mmol) in DMF was added to the sample followed by dropwise addition of 60 μL (0.34 mmol) DIPEA. Aluminum foil was used to cover the reaction vessel from light, and the mixture was stirred at room temperature for 4 h. After removal of the solvent in vacuum using CentriVap (Labconco, MO, USA), the remaining oily liquid was triturated with 0.1% TFA to afford solid compound as described by previous studies (Nagy et al., 1996). The solid was then collected by centrifugation and washed with cold diethyl ether twice to remove excessive Fmoc-Cl. HPLC analysis was used to examine purity of the product *N*-Fmoc-DOX.

#### 2.2.2. Synthesis of *N*-fmoc-DOX-14-*O*-hemiglutarate

Afterwards, 38 mg (0.05 mmol) *N*-Fmoc-DOX was reacted with 45 mg (0.4 mmol) glutaric anhydride in the presence of 60 μL (0.34 mmol) DIPEA in 5 mL DMF for 16 h under nitrogen atmosphere. The crude product *N*-Fmoc-DOX-14-*O*-hemiglutarate was purified using preparative HPLC. Specifically, the purification was performed on an XBridge^®^Prep OBD^TM^ C18 column (5 μm, 19 × 150 mm; Waters, Milford, MA, USA) at room temperature. The mobile phase is consisted of solvent A (0.01% FA in water) and solvent B (ACN). A linear gradient with a flow rate of 10 mL/min was applied in the following manner: B 0 min (30%) → 15 min (98%) → 18 min (30%) → 20 min (30%). The injection volume was 3 mL. The detection wavelength was set to 490 nm. Fractions were collected, and the eluent was evaporated to dryness.

#### 2.2.3. Synthesis of MAHNP-DOX

*N*-Fmoc-DOX-14-*O*-hemiglutarate (0.9 mg, 1 μmol), HATU (0.6 mg, 1.5 μmol) and DIPEA (1.4 μL, 8 μmol) were first mixed in 400 μL DMF for 15 min to preactivate the carboxylic group of *N*-Fmoc-DOX-14-*O*-hemiglutarate under nitrogen atmosphere. After addition of GPLGLAGDDYCDGFYACYMDV-NH_2_ (2.3 mg, 1 μmol) dissolved in 200 μL DMF, the mixture was stirred overnight in the absence of light. After removal of the solvent in vacuum using the method described earlier (Nagy et al., 1996), the residual oil was treated with 400 μL ethyl acetate. To remove the Fmoc group, 600 μL piperidine in DMF (10% *v/v*) was added and stirred for 30 min. Then the reaction was terminated by adding drops of TFA solution in DMF (10% *v/v*) about 600 μL until the solution color turned to light red. The solvent was removed in vacuum. HPLC was employed for purification.

### 2.3. Cell culture

BT474 cells (The Cell Bank of Type Culture Collection of Chinese Academy of Sciences, Shanghai, China) and SKBR3 cells (ATTC, Manassas, VA, USA) were respectively cultured in RPMI 1640 medium and McCoy’s 5 A modified medium supplemented with 10% FBS, 1% penicillin/streptomycin at 37 °C and 5% CO_2_. Fibroblast cells NIH-3T3 (ATTC, Manassas, VA, USA) were cultured in DMEM supplemented with 10% FBS. MCF-10 A cells (ATTC, Manassas, VA, USA) were maintained routinely in MEGM media supplemented with 100 ng/mL cholera toxin and 1% penicillin/streptomycin. Cells were counted with a hemacytometer (Qiujing, Shanghai, China). Cell viability was assessed by trypan blue (0.4%) exclusion, which was completed by mixing cell suspension, trypan blue and 1 × PBS in a ratio of 2:5:3 and the percentage of viable cells was counted following 5 min incubation at 37 °C.

### 2.4. Enzymatic cleavage of MAHNP-DOX by MMP-2

NIH-3T3 cells that can secrete active MMP-2 (Figure S1) were cultured in serum-free medium for 24 h. Then, 4 mL of the medium containing active MMP-2 (i.e. conditioned medium) was collected and concentrated to 500 μL using Amicon Ultra-4 10 K centrifugal filter devices (Millipore, Bedford, MA, USA) (Aye et al., 2004). Dilution of MAHNP-DOX at a concentration of 5 μM was prepared with the concentrated conditioned medium and incubated at 37 °C. Aliquots were removed at 1 h and 2 h and then analyzed by LC–MS/MS (van Duijnhoven et al., 2011). The analysis was performed on XBridge™ C18 column (5 μm, 4.6 × 250 mm; Waters, Milford, MA, USA) at room temperature. The flow rate of HPLC was 0.3 mL/min with a mobile phase consisted of solvent A (water with 0.1% FA) and solvent B (ACN). The gradient was as follows: B 0 min (10%) → 15 min (90%) → 20 min (90%) → 25 min (10%).

### 2.5. MTT assay

The cytotoxicity of MAHNP-DOX was determined using MTT test. Free DOX was used as positive control, and the cells treated with conditioned medium only were considered as negative control. BT474 and SKBR3 cells in exponential growth were seeded in a 96-well plate and incubated for 24 h at 37 °C. Then, a series of dilutions of free DOX and MAHNP-DOX at equivalent concentration were prepared with the conditioned medium and incubated for another 48 h. After an addition of MTT solution (5 mg/ml in PBS) to each well, the cells were incubated for 4 h and lysed in DMSO. Absorbance was determined at 490 nm using an EL ×800 absorbance microplate reader (Biotek, Winooski, VT, USA). The IC_50_ values were calculated from the linear regression of the dose–log response curves using GraphPad Prism 6 software (GraphPad Software, San Diego, CA, USA).

To further confirm the dependence of cytotoxicity on MMP-2 activity, NIH-3T3 cells were pre-incubated with 10 μM MMP-2 inhibitor I in serum-free medium for 24 h, and the medium was also collected termed as treated conditioned medium. Following the procedure above, the cytotoxicity of MAHNP-DOX was also determined using this medium.

### 2.6. Determination of drug distribution using confocal microscopy and HPLC

BT474 and SKBR3 cells were treated with free DOX and MAHNP-DOX in the conditioned medium at 37 °C for 1, 4, 8 and 12 h as well as MAHNP-DOX in the treated conditioned medium, respectively. Then, the cells were washed with PBS. Finally, the cells were imaged using laser confocal microscopy (Carl Zeiss, LSM 710, Oberkochen, Germany). The intracellular accumulation of drugs in nucleus was also examined by HPLC.

### 2.7. Terminal deoxynucleotidyl transferase-mediated nick-end labeling (TUNEL) assay

Transferase-mediated nick-end labeling assay was carried out using the DeadEnd Fluorometric TUNEL system kit (Promega, Madison, WI, USA). In general, apoptotic cells exhibit a strong nuclear green fluorescence at 520 nm, whereas the cells stained with DAPI exhibit a strong blue fluorescence at 620 nm. Images were acquired at room temperature with an inverted fluorescence microscope (Leica, DMI3000B, Wetzlar, Germany).

### 2.8. Inhibition of HER2 signaling

BT474 and SKBR3 cells were seeded on six well plates and exposed to free DOX and MAHNP-DOX in the conditioned medium for 24 h. The procedure of fractionation and protein extraction has been described previously (Xu et al., 2012). In detail, the cells were washed twice with ice-cold PBS and 100 μL of RIPA lysis buffer (Beyotime Institute of Biotechnology, Haimen, China) containing a protease inhibitor cocktail (Sigma-Aldrich, St. Louis, MO, USA) and a phosphatase inhibitor cocktail (Sigma-Aldrich, St. Louis, MO, USA). Then, the cell lysate was collected using cell scraper (Corning, NY, USA) and subject to Western blotting (Takeda & Xu, 2015). The membrane was incubated with anti-HER2 or anti-phospho-HER2/Tyr1248 antibody and anti-AKT or anti-phospho-AKT/Ser473 antibody (Cell Signaling Technology, Danvers, MA, USA) overnight at 4 °C followed by HRP-conjugated goat anti rabbit/mouse IgG (Beyotime Institute of Biotechnology, Jiangsu, China). Finally, proteins were detected using enhanced chemiluminescence reagent (Pierce, Rockford, IL, USA) according to the manufacturer’s protocol.

### 2.9. Cell cycle arrest

For cell cycle arrest analysis, BT474 and SKBR3 cells were seeded in a six-well plate and exposed to AHNP, free DOX and MAHNP-DOX in the conditioned medium for 48 h. Then cells were dispensed and fixed with 70% ethanol overnight at 4 °C. The cells were washed with PBS and resuspended in 400 μL of PI/RNase staining buffer (Becton-Dickinson, San Diego, CA, USA), incubated in the dark for 30 min, followed by counting the cells on a FACScalibur (Becton Dickinson, San Jose, CA, USA). The results were analyzed using Cell Quest (Becton Dickinson, San Jose, CA, USA) software.

### 2.10. *In vivo* study

Eighteen athymic nude mice (Balb/c *nu/nu*, females) were used. All animal experiments were carried out in accordance with guidelines evaluated and approved by the ethics committee of Nanjing Medical University. BT474 cells were first collected in a concentration of 1 × 10^7^/mL and orthotopically injected into the mammary fat pad area of each mouse with 0.1 mL PBS/mouse.

When the tumor size reached a volume of about 50 mm^3^, the mice were randomly divided into three groups (each group consisted of 6 mice) for the treatment with 5 mg DOX equivalent/kg of either free DOX or MAHNP-DOX once every seven days for three times. Saline was used as negative control. The animals’ weight and tumor dimensions were measured every five days. Tumor volume was calculated using an equation: TV =1/2 × *a* × *b*^2^, where *a* is the largest diameter and *b* is the smallest diameter. Each animal was tagged in the ear and followed individually throughout the experiment. For *in vivo* imaging study, the mice were intravenously injected through the caudal vein by 5 mg DOX equivalent/kg of free DOX and MAHNP-DOX, images were taken on IVIS LuminaXR imaging system (Perkin Elmer, San Diego, CA, USA) at 4 h post injection. Tumors and organ samples including heart, liver, spleen, kidneys and lungs were carefully removed and stored in liquid nitrogen. To assess the pharmacokinetic profile of the drugs, free DOX and MAHNP-DOX were injected intravenously into the mice, respectively. Blood samples were collected at 0.083, 0.25, 0.5, 1, 2, 4, 8, 12 and 24 h post injection.

### 2.11. Preparation of *in vivo* samples and biodistribution study using LC–MS/MS

All tissue samples were processed immediately after thawing and maintained in an ice-bath throughout the procedure. Approximately 50 mg tissue was weighted and resuspended in 0.2 M disodium hydrogen phosphate. Then, samples were homogenized using a Bio-Gen PRO200 homogenizer (PRO Scientific Inc., Oxford, CT, USA) for 1 min and vortexed for 5 min. 4 mL extraction solvent (9:1 chloroform:heptanol) were added. Afterward, the sample was centrifuged at 4000 rpm for 10 min. After a removal of organic layer, the remaining aqueous phase was re-extracted with 1 mL of extraction solvent for two more times. The solutions were combined and evaporated to dryness, and then resuspended in 100 mL of acetonitrile:water 50:50. For blood samples, they were centrifuged at 2000*g* for 10 min and the supernatant was collected followed by an addition of ACN three times the volume thereof. After centrifugation, the collected samples were extracted using the procedure described above.

An Agilent Series 1200 HPLC system (Agilent Technologies, Waldbronn, Germany) and a 6410 Triple Quad LC/MS mass spectrometer (Agilent Technologies, Santa Clara, CA, USA) were used. Calibration standards and QC standards of drugs were prepared in blank tissue homogenate/plasma. Daunorubicin (DAU) was used as internal standard. The chromatographic and mass spectrometric parameters have been described elsewhere (Xu et al., 2012).

### 2.12. Bliss synergy calculations

The Bliss independence (BI) model is used to define the effect of two drugs assumed to act through independent mechanisms (Buck et al., 2006; Guertin et al., 2012). BI is described by the equation *E_i_*= (*E_A_* + *E_B_*) − (*E_A_*×*E_B_*), where *E_i_* is the predicted effect (fractional inhibition in this study) by the combination of drugs *A* and *B* if they were to act additively and independently, and *E_A_* and *E_B_* are the observed effects of each drug alone. When the observed inhibition exceeds the predicted inhibition, the two compounds are considered to act synergistically.

## 3. Results

### 3.1. Synthesis and characterization of MAHNP-DOX

**Figure 2.**
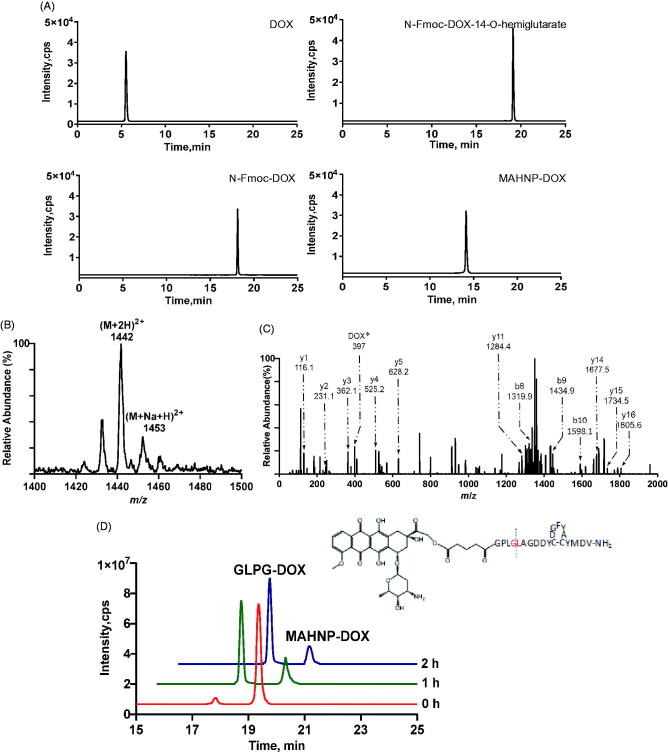
Comprehensive characterization of MAHNP-DOX. (A) The HPLC chromatograms of free DOX, *N*-Fmoc-DOX, *N*-Fmoc-DOX-14-*O*-hemiglutarate and MAHNP-DOX. The separation was performed on an XBridge®Prep OBD^TM^ C18 column (5 μm, 19 × 150 mm; Waters, Milford, MA, USA) at room temperature. The flow rate was 0.3 mL/min with a mobile phase consisted of solvent A (water with 0.1% FA) and solvent B (ACN). The gradient was as follows: B 0 min (10%) → 15 min (90%) → 20 min (90%) → 25 min (10%). (B) The parent ion spectrum of MAHNP-DOX. The mass spectrometer was interfaced with an electrospray ion source and operated in the positive mode. Q1 and Q3 were both set at unit resolution. The flow of the drying gas was 10 L/min, while the drying gas temperature was held at 350 °C. The electrospray capillary voltage was optimized to 4000 V. The nebulizer pressure was set to 45 psi. The data were collected and processed using the Agilent MassHunter Workstation Software (version B.01.04). (C) The product ion spectrum of MAHNP-DOX. The collision energy was set at 30 eV. *=unique product ions of DOX. (D) HPLC profile of MAHNP-DOX cleavage after the treatment of conditioned medium from NIH-3T3 cells. The analysis was performed on XBridge™ C18 column (5 μm, 4.6 × 250 mm; Waters, Milford, MA, USA) at room temperature. The flow rate of HPLC was 0.3 mL/min with a mobile phase consisted of solvent A (water with 0.1% FA) and solvent B (ACN). The gradient was as follows: B 0 min (10%) →15 min (90%) →20 min (90%) →25 min (10%).

As mentioned earlier, the peptide component of the peptide-DOX conjugate MAHNP-DOX is composed of the cleavable linker GPLGLAGDD and the anti-HER2 peptide AHNP, followed by conjugation with DOX (Figure 1). To avoid an undesired coupling reaction between the amino group on the daunosamine moiety of DOX and the peptide, we first prepared *N*-Fmoc-DOX by protecting the amino group with Fmoc-Cl (Nagy et al., 1996). Afterwards, *N*-Fmoc-DOX was reacted with glutaric anhydride in the presence of DIPEA to produce the intermediate *N*-Fmoc-DOX-14-*O*-hemiglutarate. Finally, the peptide was conjugated to *N*-Fmoc-DOX-14-*O*-hemiglutarate, and the Fmoc groups were removed with piperidine treatment to obtain MAHNP-DOX with 43.5% yield and 95.1% purity (Figure 2(A)). The peaks occurred at 5.5 min for DOX, at 18.2 min for *N*-Fmoc-DOX, at 19.2 min for *N*-Fmoc-DOX-14-*O*-hemiglutarate and at 14.1 min for MAHNP-DOX.

To further confirm the newly synthesized product, its molecular ion peak was obtained using a precursor ion scan. The spectrum demonstrated the presence of *m/z* 1442 for MAHNP-DOX and *m/z* 1453 for the sodium adduct of MAHNP-DOX (Figure 2(B)). Furthermore, intermediate products *N*-Fmoc-DOX (calcd, 766.2; found, 790.7 [M + Na]^+^) and *N*-Fmoc-DOX-14-*O*-hemiglutarate (calcd, 880.2; found, 902.5 [M + Na]^+^) were also monitored and their molecular weight agreed with the theoretical weights (data not shown) (Soudy et al., 2013). Given that these molecular ion peaks cannot offer the structural information for molecules (Sheng et al., 2015), tandem mass spectrometry and collision-induced dissociation (CID) were performed, and the product ion spectrum was resolved based on the similarity between the peptide-drug conjugate fragmentation and fragmentation of the individual peptide and conjugated drug. As shown in Figure 2(C), some characteristic sequence-specific b ions and y ions resulting from the cleavage of the peptide backbone in the positive ion mode were observed. In addition, the specific product ion of DOX (e.g. *m/z* 397) is present in the spectrum.

The sensitivity of MAHNP-DOX to MMP-2 was studied using HPLC. To date, there has been substantial evidence indicating stronger signals for MMP-2 in stromal cells compared to cancer cells, while stromal fibroblasts interact with cancer cells by secreting and activating MMP-2 (Saad et al., 2002; Hassona et al., 2014). Thus, a concentrated fibroblast-conditioned medium was used in this study to mimic the tumor microenvironment to some extent (Aye et al., 2004). Theoretically, MAHNP-DOX can be cleaved between glycine (G) and leucine (L) residues by MMP-2. Thus, the peaks for MAHNP-DOX and one product after cleavage of GLPG-DOX were monitored. As shown in Figure 2(D), the peaks occurred at 19.4 and 17.8 min, respectively. The identity of GLPG-DOX was also validated using LC-MS (Figure S2). Finally, the efficiency of cleavage can achieve 80.8% after 2 h of incubation. Notably, all of the *in vitro* experiments were performed in the conditioned medium if the condition is not specified.

### 3.2. *In vitro* cytotoxicity of MAHNP-DOX

**Figure 3.**
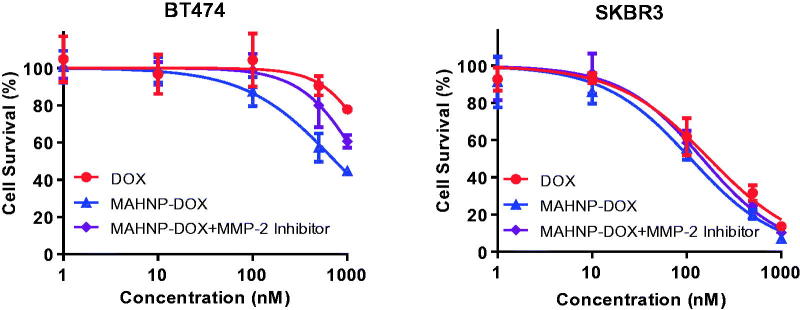
Cytotoxicity profiles of free DOX, MAHNP-DOX and MAHNP-DOX with MMP-2 inhibitor pretreatment of BT474 and SKBR3 cells (*n* = 3).

To compare the cytotoxic effects of MAHNP-DOX with free DOX, we employed two cell lines (i.e. BT474 and SKBR3). The HER2 overexpression in these cells was confirmed by Western blotting (Figure S3). The cells were incubated with various concentrations of free DOX and MAHNP-DOX individually. The cell viability was evaluated using the MTT method. As shown in Figure 3, MAHNP-DOX inhibited the growth of BT474 and SKBR3 cells in a dose-dependent manner (*n* = 3), as well as free DOX. The IC_50_ values of MAHNP-DOX for BT474 and SKBR3 cells calculated from the dose-log response curves were 746.8 ± 81.5 nM and 110.1 ± 12.7 nM, respectively. These values were significantly lower than those of free DOX (2075.0 ± 368.0 for BT474 cells and 172.9 ± 19.2 for SKBR3 cells, *p* < .01).

To classify the effect of the DOX and AHNP combination, the Bliss independence method was used. The result indicates that MAHNP-DOX (observed) was more effective than either agent alone. Additionally, more fractional inhibition by MAHNP-DOX was observed compared to the predicted inhibition by simple combination of DOX and AHNP in both BT474 cells and SKBR3 cells, which suggested a synergistic interaction between DOX and AHNP (Table S1).

In addition to free DOX and AHNP, the cytotoxicity of MAHNP-DOX also depends on MMP-2 activity. To illustrate this dependence, BT474 and SKBR3 cells were exposed to MAHNP-DOX in the conditioned medium from NIH-3T3 cells pretreated with MMP-2 inhibitor (*n* = 3) (Figure 3). The obtained IC_50_ values were 1348.0 ± 153.7 nM for BT474 cells and 146.7 ± 16.3 nM for SKBR3 cells, which were significantly higher compared to the values obtained without inhibitor pretreatment (*p* < .01).

### 3.3. Effects of MAHNP-DOX on apoptosis and DNA damage

Nuclei containing DNA damage were detected by TUNEL assay, and the corresponding BT474 and SKBR3 cells were counterstained with DAPI to show the nuclei. Fluorescence microscopy showed that the number of TUNEL-positive cells significantly increased after 24 h treatment with both free DOX and MAHNP-DOX (Figure 4). For BT474 cells, the percentage of apoptotic cells was 55.1 ± 3.2% for MAHNP-DOX and greater than 34.2 ± 2.1% for free DOX (*n* = 3, *p* < .01, Figure 4(A)). Similarly, SKBR3 cells had more apoptotic cells (60.0 ± 3.9%) after treatment with MAHNP-DOX compared to free DOX (44.8 ± 2.8%; *n* = 3, *p* < .05, Figure 4(B)).

Figure 4.Cellular apoptosis of (A) BT474 and (B) SKBR3 cells detected using TUNEL assay. Nuclei were stained with DAPI, and merged images were considered as apoptotic cells. Fluorescence images of (C) BT474 and (D) SKBR3 cells after the treatment with free DOX and MAHNP-DOX and (E) the measured DOX amounts in the nuclei isolated from the cells (*n* = 3). The amounts were averaged on the number of cells. Two-tailed Student’s *t*-test was used. **p < .01.

**Figure:**
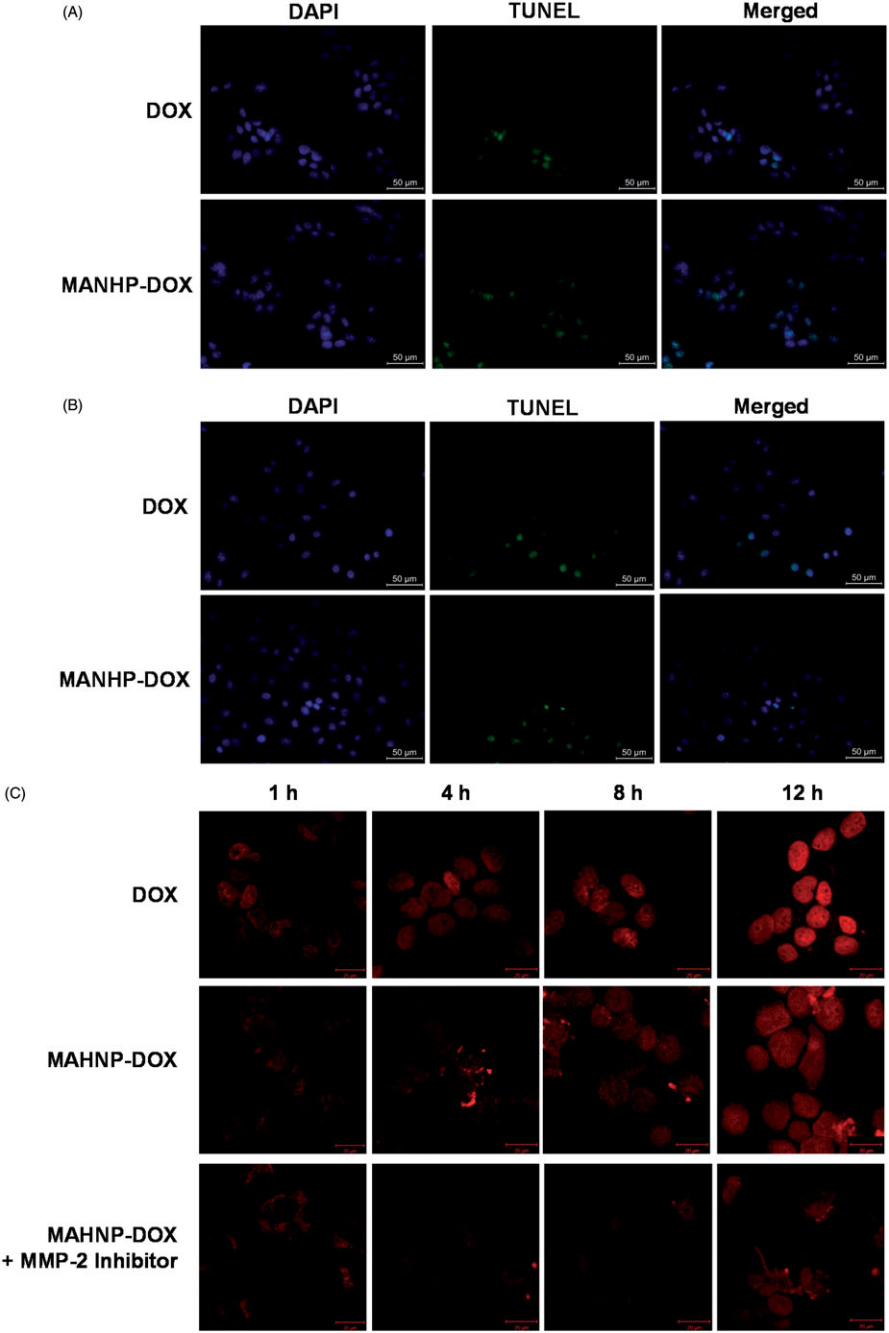

**Figure:**
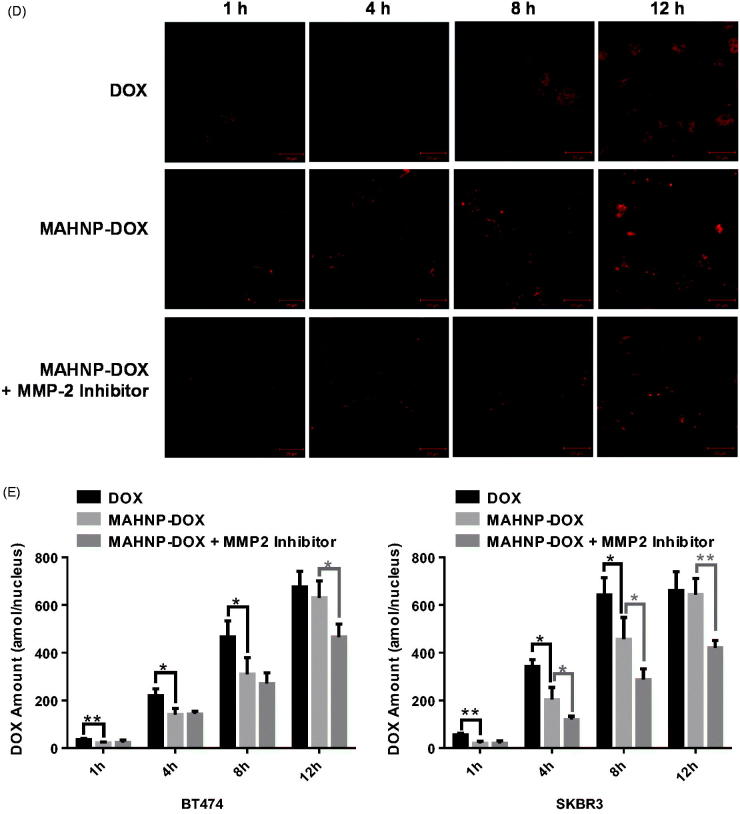

### 3.4. Intracellular drug accumulation

Doxorubicin accumulation in cells was imaged after 1, 4, 8 and 12 h treatment with DOX and MAHNP-DOX in BT474 and SKBR3 cells (Figure 4(C, D)). Following a procedure that was described previously (Xu et al., 2012), the amounts of DOX in nuclei were also determined using HPLC (Figure 4(E)). The mechanism for MAHNP-DOX accumulation in cells and nuclei should be similar to the mechanism for free DOX because this peptide-conjugated DOX can be cleaved into free DOX by MMP-2 that is mainly secreted by stromal cells and distributed abundantly around tumor cells in the extracellular matrix (Jodele et al., 2006). Afterward, free DOX penetrates the cell membrane through simple diffusion (Kojima et al., 2013) followed by translocation to the nucleus (Kiyomiya et al., 2001; Minotti et al., 2004). The observed difference in the distribution patterns between MAHNP-DOX and DOX indicated there was MMP-2-dependent drug release (Kiyomiya et al., 2001; Minotti et al., 2004). Furthermore, DOX accumulation in the nuclei was sustained when the cells were pre-incubated with the MMP-2 inhibitor (Figure 4(C, D)), which also provided evidence for the involvement of MMP-2.

The tumor targeting of MAHNP-DOX was also confirmed by significantly lower accumulation and a higher IC_50_ in normal MCF-10 A breast cells, which are HER2 negative and normally have MMP-2 mostly in its inactive form (Figure S4) (Lee et al., 1996).

### 3.5. Effect of MAHNP-DOX on the HER2 signaling pathway

Even though synergistic effects of the AHNP and DOX combination have been observed, the mechanism underlying these effects is still unknown. It is well known that DOX is cytotoxic; however, it can also induce the activation of the HER2/PI3K/AKT signaling cascade, which is associated with cell growth and proliferation in various cancers (Milosevic et al., 2014). Interestingly, much evidence has suggested that the peptide AHNP can inhibit the HER2/PI3K/AKT signaling as well as trastuzumab (Fantin et al., 2005). Therefore, the effect of MAHNP-DOX on the HER2/PI3K/AKT signaling pathway was investigated in this study. After the treatment of BT474 cells with DOX and MAHNP-DOX for 24 h (Figure S5), proteins were extracted and analyzed for the expression of HER2, AKT and their respective active forms (i.e. phospho-HER2 and phospho-AKT). As a result, phospho-HER2 and phospho-AKT increased after treatment with DOX, which correlated with the results of several previous studies (Li et al., 2007; Smolensky et al., 2015). By comparison, a significant decrease in these active forms was found in cells treated with MAHNP-DOX.

### 3.6. Effect of MAHNP-DOX on cell cycle arrest

Previous studies have demonstrated that trastuzumab can lead to cell arrest during the G1 phase (Le et al., 2003; Ménard et al., 2003; Gemmete & Mukherji, 2011). To determine whether the newly synthesized conjugate has a similar property, a cell cycle assay was performed using flow cytometry on BT474 and SKBR cells after 48 h of drug exposure. As shown in Figure S6, the peptide AHNP (10 μM) alone increased cell population in the G1 phase by 6.58% and 9.95% for BT474 and SKBR3 cells, respectively. While DOX is most active in the S phase, MAHNP-DOX can cause an increase in the cell population in the G1 phase compared to DOX alone, but the increase is lower than the increase for the peptide alone.

### 3.7. *In vivo* anti-tumor effect and biodistribution study

**Figure 5.**
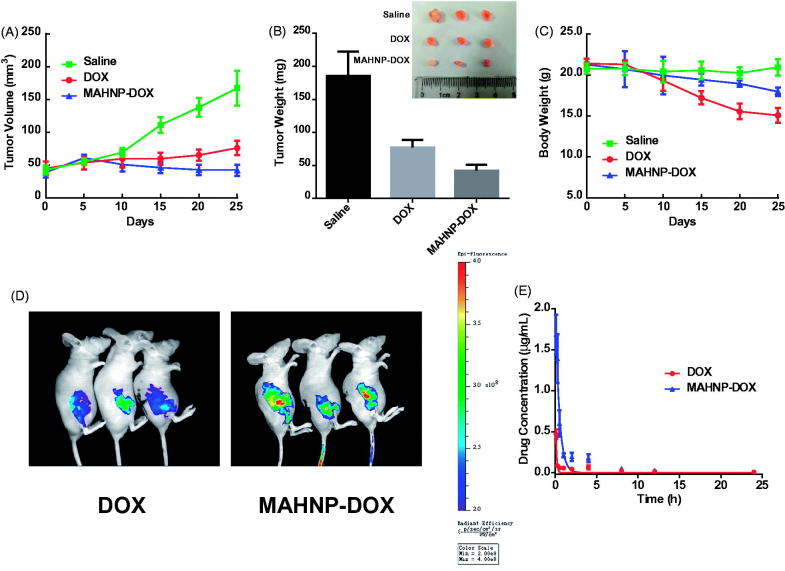
(A) Tumor volume change, (B) tumor weight and excised tumor on day 25 and (C) body weight change of mice with BT474 xenografts after the treatment with free DOX, MAHNP-DOX and saline. (D) *In vivo* images of mice and (E) drug concentration profiles in plasma (*n* = 3) are also shown. Two-tailed Student’s *t*-test was used. *p < .05, **p < .01.

In the *in vivo* experiment, BT474 tumor-bearing mice were first treated with MAHNP-DOX, free DOX and saline. Figure 5(A) shows tumor growth curves for all the groups. On day 25 after treatment, MAHNP-DOX and free DOX inhibited tumor growth by 74.7 ± 5.1% and 53.4 ± 6.6%, respectively. In addition, MAHNP-DOX had a significant tumor weight inhibition that was 45.4 ± 11.2% higher compared to that of free DOX (Figure 5(B)). Toxicity was observed in mice treated with free DOX, whereas mice treated with MAHNP-DOX had less weight loss (Figure 5(C)).

**Figure 6.**
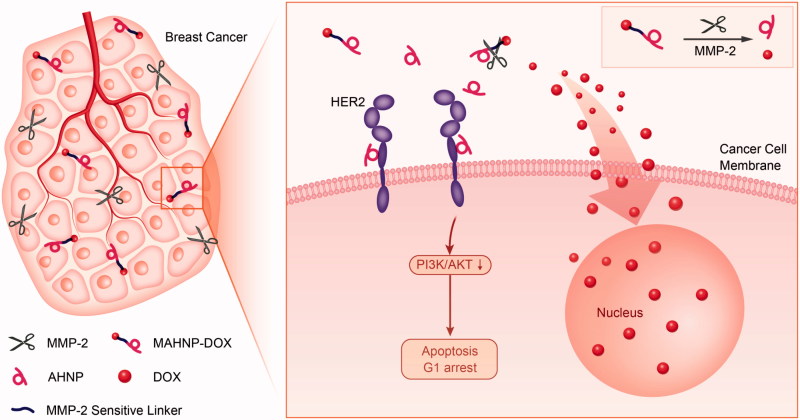
Molecular mechanism of MAHNP-DOX. MAHNP-DOX is cleaved extracellularly by MMP-2 and then DOX enters the cells, while the anti-HER2 peptide AHNP binds to HER2, inhibiting the HER2/PI3K/AKT signaling pathway and leading to cell arrest during G1 phase.

Then, fluorescent images were taken at 4 h after intravenous injection. As shown in Figure 5(D), the drug amount in the tumors of mice treated with MAHNP-DOX was significantly higher than that of free DOX (*p* < .01). This result was further confirmed by HPLC (Figure S7). Furthermore, drug accumulation in heart, liver, spleen, lung and kidney was lower than that in the mice treated with free DOX (*p* < .05, Figure S8). This observation was also consistent with the HPLC results. Additionally, TUNEL staining indicated more apoptotic cells at the tumor site in the MAHNP-DOX group as compared to the free DOX group, while apoptosis in other organs reduced significantly in the MAHNP-DOX group (Figure S9).

Finally, drug concentration profiles in plasma after treatment with MAHNP-DOX and free DOX were evaluated and the pharmacokinetic parameters were calculated (*n* = 3) (Figure 5(E)). Compared to free DOX, MAHNP-DOX showed a longer half-life t_1/2_ in plasma (17.6 ± 6.4 h *vs.* 7.7 ± 1.6 h) and cleared much more slowly from the body (4640.2 ± 2679.9 mL/h/kg *vs.* 1669.8 ± 1259.3 mL/h/kg).

## 4. Discussion

Doxorubicin is a widely used anthracycline that has been shown to be highly effective against a variety of human malignancies, including breast cancer. From a clinical perspective, the use of DOX could be limited due to its low tumor selectivity and the reduced sensitivity during treatment. Thus, new efforts are needed to improve DOX efficacy and safety. In this context, combination drugs that affect multiple targets may be a promising strategy.

Recent developments in biological systems and the overall clinical experience have revealed that single-target drugs may not always induce the desired effect on the entire biological system, even if they successfully inhibit or activate a specific target (Lu et al., 2012). One possible reason is that organisms can affect effectiveness through compensation. The development of diseases, particularly complex ones, involves several aspects. For DOX, the best-known mechanism is that it interacts with topoisomerase II (TOPOII) and induces apoptosis (Tewey et al., 1984). However, other mechanisms of DOX remain unclear. A potential mechanism is that DOX can activate the PI3K/AKT pathway, which is responsible for the reduced sensitivity of DOX in the treatment. Thus, scientists have proposed multi-target drug design to achieve the desired treatment. The success of a previous DOX and trastuzumab combination led to two targets (i.e. TOPOII and HER2) being chosen in a new design. Through this approach, the activation of the HER2/PI3K/AKT signaling pathway by DOX can be compensated for.

Most multi-target therapeutics have been developed as a mixture of agents with selectivity for individual targets, but in some cases multi-target action was built into a single chemical entity and achieves therapeutic benefit (Morphy et al., 2004), such as trastuzumab-DOX. However, one of the major issues for ADC drugs is their molecular size and the degree of tumor penetration. To optimize drug delivery, a peptide was usually used instead of a protein. One feature of this strategy is the ease of synthesis and the controlled drug-to-peptide ratio. To date, various linkages, such as hydrazone, oxime and ester bonds were employed to form a DOX-peptide conjugate (Schlage et al., 2011). Among these linkages, a Michael-addition of a thiol to a maleimide is one of the most common classes in conjugation (Sann, 2006), which has also been used in our previous study (Sheng et al., 2015; Yuan et al., 2016). Theoretically, this reaction is specific and efficient. However, a side reaction called a thiol-disulfide interchange can occur in the presence of disulfide bond, which leads a thiol to react with a disulfide (R'SSR') to form a new disulfide (RSSR') and a thiol (R'SH) derived from the original disulfide (Singh & Whitesides, 1993). Thus, glutaric anhydride was employed to form an ester bond-linked conjugate in this study (Schlage et al., 2011).

In this study, the efficacy of MAHNP-DOX was evaluated and compared with that of free DOX using HER2-positive human breast cancer cells BT474 and SKBR3 as *in vitro* model systems and nude mice with BT474 xenografts as an *in vivo* model. The *in vivo* results are in agreement with those of *in vitro*. In brief, MAHNP-DOX can specifically target breast cancer cells and tumors compared to free DOX according to fluorescent images and HPLC results. TUNEL result also indicated that more apoptotic cells were associated with the involvement of MAHNP. In addition, MAHNP-DOX demonstrated a much lower *in vitro* IC_50_, and its *in vivo* extent of inhibition in mice was more evident compared to free DOX. It should be noted that most of the peptides themselves do not have a significant cytotoxic function, but only a targeting function. In comparison, AHNP has both targeting and cytotoxic functions (Park et al., 2000). However, its targeting property had a more important role in normal cells and tissue where HER2 expression is low and either HER2 activation or DOX delivery *via* potential receptor-mediated endocytosis could be substantially circumvented. In this study, the selectivity and cytotoxicity of MAHNP-DOX for cancer cells and tumors is mainly realized by the MMP-2 sensitive peptide linker.

As previously reported, the direct binding of drugs and peptides should be avoided because it could result in complete loss of drug activity. Several early studies suggested that it was better for cytotoxic activity that the drug was liberated from the conjugate where it is specifically required (Sann, 2006). Therefore, most current peptide-drug conjugates are linked by cleavable structures. The linker structures should enable a specific release of drugs in a tumor microenvironment, which exhibits some altered properties compared to a normal cellular environment, such as lower pH, over-expressed or differentially expressed enzymes, altered redox potential and hyperthermia and then the released drugs can exert their effects (He et al., 2016). Therefore, enzyme-triggered drug release is one of the most popular strategies for drug delivery systems (Torchilin, 2014; Chen et al., 2017).

MMP-2, which mediates the degradation of the extracellular matrix (ECM), is known to be is one of the most important enzymes that is considered to facilitate tumor invasion in a series of invasive breast carcinomas, which is overexpressed in a variety of malignant tissues compared to normal tissues, such as cancers of the breast, colon, stomach and lung. The majority of MMP-2 is secreted from the tumor stroma. The strategy in this study can lead to DOX and anti-HER2 peptide release outside of tumor cells, and then both can act on their targets.

## 5. Conclusions

In this study, a peptide-DOX conjugate (MAHNP-DOX) was designed and successfully synthesized. The hybrid peptide was composed of a cleavable linker GPLGLAGDD and the anti-HER2 peptide AHNP, and then it was conjugated covalently with a DOX derivate (*N*-Fmoc-Dox-14-*O*-hemiglutarate). The obtained peptide-DOX conjugate can effectively target HER2-positive breast cancers and deliver DOX to nuclei faster due to the release of free DOX in the extracellular matrix through cleavage of MMP-2. Furthermore, the HER2 targeting peptide AHNP displays similar activity to that of trastuzumab, which enhanced the cytotoxicity of DOX by inhibiting HER2 signaling, especially downstream PI3K-AKT signaling, and inducing G1 arrest. With a combination of AHNP and DOX, the peptide-DOX conjugate MAHNP-DOX could produce a greater level of efficacy and safety in a synergistic manner. In near future, extensive preclinical toxicological and pharmacological studies of MAHNP-DOX will be performed in our lab to support further clinical trials. Finally, the therapeutic potential of MAHNP-DOX implied that multi-target drugs with a rational design could produce better therapeutic effects.

## Supplementary Material

IDRD_Chen_et_al_Supplemental_Content.docx
