# Supplementary material for: Doxorubicin conjugated with a trastuzumab epitope and an MMP-2 sensitive peptide linker for the treatment of HER2-positive breast cancer
Source: Drug Deliv. 2018 Feb 6;25(1):448–60. doi: 10.1080/10717544.2018.1435746 (PMC6058718; doi:10.1080/10717544.2018.1435746)
Supplement: IDRD_Chen_et_al_Supplemental_Content.docx [file IDRD_A_1435746_SM2933.docx]

**Supporting Information For**

**Doxorubicin Conjugated with a Trastuzumab Epitope and an MMP-2 Sensitive Peptide Linker for the Treatment of HER2-positive Breast Cancer**

*Yiwen You, Zhiyuan Xu, Yun Chen**

*** Dr. Yun Chen, School of Pharmacy, Nanjing Medical University, 818 Tian Yuan East Road, Nanjing, 211166, China and China State Key Laboratory of Reproductive Medicine, Nanjing, 210029, China

**Table of Contents:**

Table 1S. Sensitivity of cell lines to the combination of AHNP and DOX.

Figure 1S. Western blot analysis of concentrated, serum-free cultured medium.

Figure 2S. The parent ion spectrum of GLPG-DOX.

Figure 3S. Western blotting of HER2 in cells.

Figure 4S. MAHNP-DOX accumulation in cells.

Figure 5S. Phosphorylation of HER2 and AKT after addition of MAHNP-DOX and free DOX.

Figure 6S. Cell cycle arrest induced by AHNP, DOX and MAHNP-DOX.

Figure 7S. The measured DOX amounts in the tumor of mice treated with DOX and MAHNP-DOX.

Figure 8S. The measured DOX amounts in the organs of mice treated with DOX and MAHNP-DOX.

Figure 9S. TUNEL staining images of the organs after the treatment of DOX and MAHNP-DOX, as well as control.

**Supplementary Tables**

Table 1S. Sensitivity of cell lines to the combination of AHNP and DOX. A theoretical curve was calculated for combined inhibition using the equation E_bliss_ = E_A_ +E_B_-E_A_×E_B_, where E_A_ and E_B_ are the fractional inhibitions obtained by AHNP alone and free DOX alone at 1 μM. Here, E_bliss_ is the fractional inhibition that would be expected if the combination of the two drugs was exactly additive. If the experimentally measured fractional inhibition is less than E_bliss_, the combination was said to be synergistic. If the experimentally measured fractional inhibition is greater than E_bliss_, the combination was said to be antagonistic.

**Supplementary Figures**

Figure 1S. Western blot analysis of concentrated, serum-free cultured medium from BT474, SKBR3 and NIH-373 cells

Figure 2S. The parent ion spectrum of GLPG-DOX.

Figure 3S. Western blotting of HER2 in BT474, SKBR3 and MCF-10A cells.

Figure 4S. MAHNP-DOX accumulation in MCF-10A, BT474 and SKBR3 cells and its cytotoxicity profile in MCF-10A cells.

Figure 5S. Phosphorylation of HER2 and AKT after addition of MAHNP-DOX and free DOX in BT474 and SKBR3 cells.

Figure 6S. Cell cycle arrest in BT474 and SKBR3 cells induced by AHNP, DOX and MAHNP-DOX.

Figure 7S. The measured DOX amounts in the tumor of mice treated with DOX and MAHNP-DOX.

Figure 8S. The measured DOX amounts in the organs of mice treated with DOX and MAHNP-DOX.

Figure 9S. TUNEL staining images of the organs after the treatment of DOX and MAHNP-DOX, as well as the control.
